# Efficacy and safety of the dexamethasone implant in vitrectomized and nonvitrectomized eyes with diabetic macular edema: A systematic review and meta-analysis

**DOI:** 10.3389/fphar.2022.1029584

**Published:** 2022-12-01

**Authors:** Qiongzhen Yuan, Yanling Liu, Yueqin Gou, Hanyue Xu, Yunxia Gao, Yilin Liu, Yi Chen, Ming Zhang

**Affiliations:** ^1^ Department of Ophthalmology, West China Hospital, Sichuan University, Chengdu, China; ^2^ Operating Room, West China Hospital, Sichuan University/West China School of Nursing, Sichuan University, Chengdu, China

**Keywords:** dexamethasone implant, vitrectomized, nonvitrectomized, diabetic macular edema, meta-analysis

## Abstract

**Purpose:** To compare the efficacy and safety of the intravitreal dexamethasone (DEX) implant for the treatment of diabetic macular edema (DME) in vitrectomized and nonvitrectomized eyes.

**Methods:** We performed a literature search in four electronic databases (PubMed, EMBASE, MEDLINE, and Cochrane Library) from inception to 22 May 2022. Studies comparing the efficacy of the DEX implant in vitrectomized and nonvitrectomized eyes with DME with at least 3 months of follow-up were included. The main outcomes included comparison of the mean change in the best-corrected visual acuity (BCVA) and central macular thickness (CMT) from baseline to different follow-up endpoints between the vitrectomized and nonvitrectomized groups. The secondary outcomes were the mean duration of action for the first DEX implantation and the number of required injections throughout the follow-up period. Safety data were collected and compared.

**Results:** The final analysis included 7 studies involving 582 eyes, 208 vitrectomized eyes and 374 nonvitrectomized eyes. The mean between-group differences in BCVA improvement were not significant at any endpoint, with averages difference of −0.07 logarithm of the minimum angle of resolution (logMAR) (*p* = 0.088) at 1 month, −0.03 logMAR (*p* = 0.472) 3 months, −0.07 logMAR (*p* = 0.066) 6 months, and −0.04 logMAR (*p* = 0.486) 12 months. The mean between-group differences in CMT reduction were not statistically significant, with mean differences of 7.17 μm (*p* = 0.685) at 1 month, 20.03 μm (*p* = 0.632) 3 months, −1.80 μm (*p* = 0.935) 6 months, and −25.65 μm (*p* = 0.542) 12 months. However, the vitrectomized group had a significantly shorter duration of action during the first DEX implantation than the nonvitrectomized group, with a mean difference of 0.8 months (*p* = 0.005). No significant between-group differences were detected for the number of required injections or safety profile.

**Conclusion:** This meta-analysis showed similar efficacy and safety of the sustained-release DEX intravitreal implant for vitrectomized and nonvitrectomized eyes with DME. The intravitreal DEX implant could be considered an effective choice for DME treatment in eyes with prior vitrectomy.

## 1 Introduction

Diabetic retinopathy (DR) is the most common vascular retinopathy affecting working-age individuals worldwide. Among patients with DR, diabetic macular edema (DME) is the major cause of vision deterioration (Le et al., 2021). DME, the accumulation of fluid exudation within the retinal layers around the macular area, can occur at any stage of DR (Wong et al., 2016; Tan et al., 2017). Vascular endothelial growth factor (VEGF) and inflammatory factors play important roles in DME formation.

Pars plana vitrectomy is a mainstay and beneficial surgery for the treatment of patients with DR with complicated conditions, such as vitreous hemorrhage, epiretinal membrane, vitreomacular traction, and retinal detachment. After vitrectomy, the vitreous gel is replaced with less viscous liquids, which enhance the transport of oxygen to the ischemic retina and clearance of cytokines, such as VEGF, thereby relieving macular edema (ME) and neovascularization (Stefánsson, 2009; Yoshida et al., 2010). However, due to the chronic nature of DR, many patients may develop recurrent or persistent DME after the surgical procedure, requiring subsequent intravitreal drug therapy.

Pharmacokinetic changes in vitrectomized eyes may have unfavorable effects on the efficacy and duration of intravitreal medications. Anti-VEGF agents and other intravitreal drugs (5 fluorouracil, triamcinolone, and amphotericin B) have been washed out more rapidly in vitrectomized eyes than in nonvitrectomized eyes (Doft et al., 1985; Jarus et al., 1985; Chin et al., 2005; Christoforidis et al., 2013). Although intravitreal anti-VEGF drugs have been recommended as the first-line therapy for DME, their efficacy in vitrectomized eyes is not ideal. Chen and co-workers reported greater anatomical and functional improvements, along with fewer injections in nonvitrectomized eyes than in vitrectomized eyes after injection of ranibizumab (Chen et al., 2018). Several studies have demonstrated no significant anatomical and functional improvements with bevacizumab and aflibercept in vitrectomized eyes (Yanyali et al., 2007; Okamoto et al., 2014; Chen et al., 2017).

The dexamethasone (DEX) intravitreal implant (Ozurdex; Allergan, Irvine Inc., CA, United States), a biodegradable device designed to slowly release DEX for up to 6 months after injection, has been approved for the management of DME and ME following retinal vein occlusion and noninfectious posterior uveitis (Lowder et al., 2011; Boyer et al., 2014; Wecker et al., 2021). Its efficacy and safety have been well demonstrated not only for treatment-naive DME (Boyer et al., 2014; Mathis et al., 2020) but also for persistent DME refractory to intravitreal anti-VEGF medications (Zhioua et al., 2015; Shah et al., 2016; Yuan et al., 2022). Due to the slow-release properties of the DEX implant, its duration of action and efficacy remain satisfactory in post-vitrectomy eyes. A prospective clinical trial investigated the efficacy and tolerability profiles of intravitreal DEX implantation in 55 vitrectomized eyes with DME over a 26-week period (Boyer et al., 2011). They reported significant BCVA and CMT improvement at 8 and 26 weeks after receiving a single intravitreal injection. Additionally, a retrospective study demonstrated that the DEX implant achieved significantly better anatomical/visual improvement and fewer injections than intravitreal ranibizumab in the treatment of vitrectomized eyes with DME (Wang et al., 2021).

Although several studies have compared the safety and effectiveness of the DEX intravitreal implant for treating DME in nonvitrectomized and vitrectomized eyes, no comprehensive synthesis of available data has been published. Therefore, we conducted this systematic review and meta-analysis to compare the visual and anatomical improvements of the sustained-release DEX implant in nonvitrectomized and vitrectomized eyes for DME therapy.

## 2 Materials and methods

### 2.1 Literature search

We performed a systematic search of relevant topics in 4 electronic databases (PubMed, EMBASE, MEDLINE, and Cochrane Library) from inception to 22 May 2022. The literature search strategy included a combination of the following terms: “dexamethasone,” “Ozurdex,” “vitrectomized,” “diabetic macular edema” and “DME.” Studies published in English that compared the efficacy of the DEX intravitreal implant in nonvitrectomized and vitrectomized eyes with DME were reviewed. We further investigated the references of eligible articles to find any relevant studies. We performed this meta-analysis in accordance with the Preferred Reporting Items for Systematic Reviews and Meta-Analyses (PRISMA) checklist (Liberati et al., 2009).

### 2.2 Eligibility criteria

Inclusion criteria were as follows: (1) patients with DME older than 18 years; (2) studies with a comparison of the efficacy of the DEX intravitreal implant between nonvitrectomized and vitrectomized eyes; (3) studies with follow-up for at least 3 months; and (4) the primary measures, central macular thickness (CMT) and best-corrected visual acuity (BCVA), were presented as mean ± standard deviation. Reviews, letters without data, case reports, and conference abstracts were excluded. The most recently published studies were included when the same study patients were presented in various publications.

### 2.3 Outcome measures

The primary outcomes included a comparison of the mean changes in BCVA and CMT at different follow-up endpoints (1, 3, 6, and 12 months) between the nonvitrectomized and vitrectomized groups after intravitreal DEX implant therapy. We compared the mean duration of action during the first DEX implantation and the mean number of injections required over the follow-up period between the 2 groups as secondary outcomes. We presented the BCVA data as the logarithm of the minimum angle of resolution (logMAR). Safety data, including ocular and systemic adverse events (AEs) during the follow-up period, were also collected.

### 2.4 Data extraction and quality assessment

Two independent investigators (QY and YG) conducted a full-text assessment of the included studies and extracted relevant information from each study. The following information were collected: first author, publication year, research location, number of samples (patients/eyes), mean age, number of DEX injections, follow-up duration, mean BCVA and change in CMT, duration of action, rate of elevated intraocular pressure (IOP), and other recorded AEs.

The same 2 reviewers independently assessed the quality of all included studies based on the modified Downs and Black checklist (Downs and Black, 1998). This evaluation tool is suitable for both randomized controlled trials and nonrandomized trials. The score range provides the corresponding levels of quality: excellent quality (26–28 of a maximum of 28 points), fair quality (15–19), and poor quality (0–14). The higher the score, the lower is the risk of bias. All included trials were classified as having fair quality. We consulted a third reviewer (HX) to reach a consensus in case of any discrepancies.

### 2.5 Statistical analyses

Statistical analyses were performed using STATA software (version 15.0; Stata Corporation, College Station, TX, United States). In terms of continuous data, the weighted mean difference was calculated, and the pooled results are presented as the mean difference with a 95% confidence interval (CI). Statistical heterogeneity was assessed using the Cochran Q test, along with the statistical value I^2^. Heterogeneity across studies was considered acceptable if *p* > 0.1 and I^2^ < 50%. The random-effects model was adopted even in the absence of statistically significant inter-study heterogeneity because it can provide more conservative effect estimates in the case of residual heterogeneity. Funnel plots and the Egger test were used to assess potential publication bias in all included studies. We performed sensitivity analysis based on the leave-one-out approach. A 2-sided alpha level of *p* < 0.05 was regarded to be statistically significant.

## 3 Results

### 3.1 Study selection and description of the studies

**FIGURE 1:**
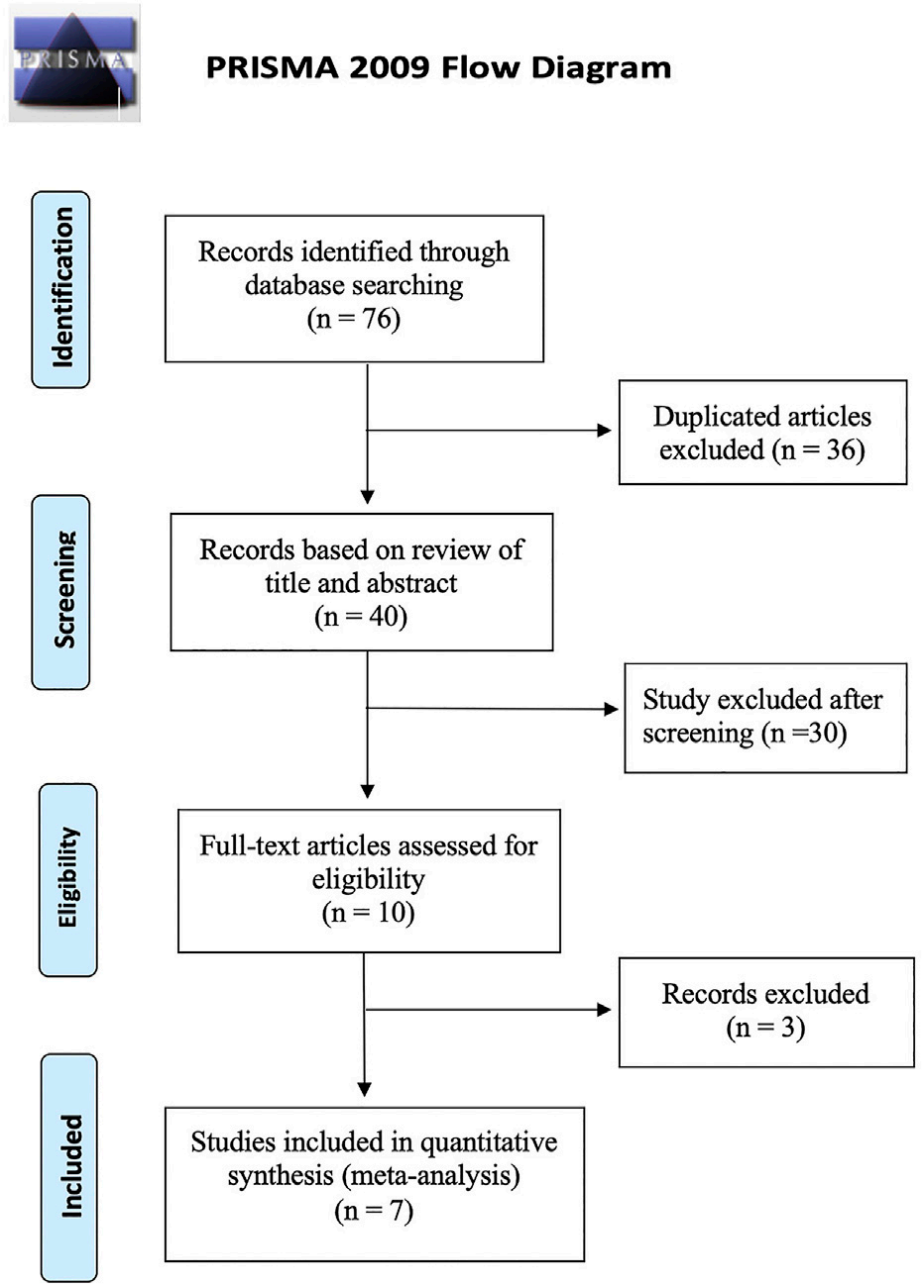
Preferred Reporting Items for Systematic Reviews and Meta-Analyses flow chart of study identification and selection process.

The study identification and selection process based on the PRISMA flow chart is presented in Figure 1. We identified 76 studies by database searching and eliminated 36 duplications. After screening titles and abstracts, 30 studies were removed because they were on improper topics or were case reports, reviews, and letters. Three publications were excluded after full-text review. Finally, 7 articles that met the criteria were included (Medeiros et al., 2014; Bonnin et al., 2015; Çevik et al., 2018; Bastakis et al., 2019; Wang et al., 2020; Iglicki et al., 2022; Kwon and Park, 2022). All eligible studies were retrospective in design, with follow-up durations ranging from 4 to 36 months.

### 3.2 Baseline characteristics

The basic characteristics of the 7 included studies are shown in Supplementary Table S1. In total, 582 eyes were included in our analyses, 208 vitrectomized and 374 non-vitrectomized eyes. The sample sizes ranged from 18 to 236 eyes, with a mean patient age ranging from 57.82 to 76 years. Average baseline BCVA values ranged from 0.57 to 0.98 logMAR in the vitrectomized group and from 0.57 to 0.88 logMAR in the nonvitrectomized group, without significant between-group difference (*p* = 0.647). Average baseline CMT values ranged from 462.19 to 635.55 μm in the vitrectomized group and from 475.11 to 640 μm in the nonvitrectomized group, without significant between-group difference (*p* = 0.905). The average number of DEX implantations ranged from 1 to 3.41 times in the vitrectomized group and from 1 to 3.54 times in the nonvitrectomized group.

### 3.3 Best-corrected visual acuity

**FIGURE 2:**
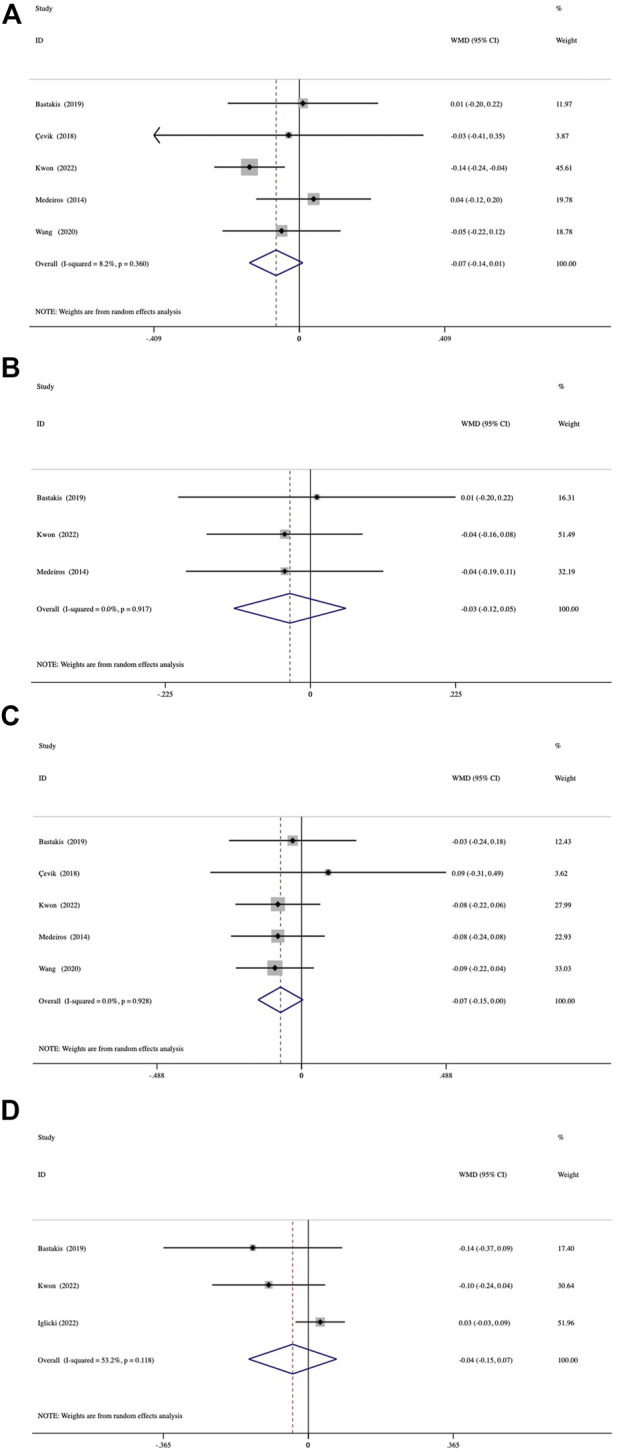
Forest plots of the comparison of mean BCVA (best-corrected visual acuity) improvement between the vitrectomized and nonvitrectomized groups **(A)** 1 month, **(B)** 3 months, **(C)** 6 months, and **(D)** 12 months.

Both groups achieved significant BCVA improvement at 1, 3, and 6 months (all, *p* < 0.05; Supplementary Table S2). At 12 months, the average BCVA gain was also significant in the vitrectomized group but not in the nonvitrectomized group (*p* = 0.059). The mean BCVA gain from baseline to the 4 follow-up visits was compared between the groups (Figure 2). The assessment of mean BCVA improvement from baseline to the first month was performed across 5 studies and showed no significant between-group differences of −0.07 logMAR (95% CI, −0.14 to 0.01; *p* = 0.088) (Figure 2A). We conducted the comparison of mean BCVA change at 3 months in 3 studies, which presented a nonsignificant difference of -0.03 logMAR (95% CI, −0.12 to 0.05; *p* = 0.472) (Figure 2B). In 5 studies followed up for 6 months, we detected no significant between-group differences in mean BCVA gain, with an average difference of −0.07 logMAR (95% CI, −0.15 to 0.00; *p* = 0.09) (Figure 2C). At 12 months, the comparison of mean BCVA change included 3 studies and demonstrated no significant between-group differences (−0.04 logMAR; 95% CI, -0.15 to 0.07; *p* = 0.486) (Figure 2D). We detected no significant inter-study heterogeneity between the studies at 1 month (I^2^ = 8.2%, *p* = 0.360), 3 months (I^2^ = 0%, *p* = 0.917), or 6 months (I^2^ = 0%, *p* = 0.928).

### 3.4 Central macular thickness

**FIGURE 3:**
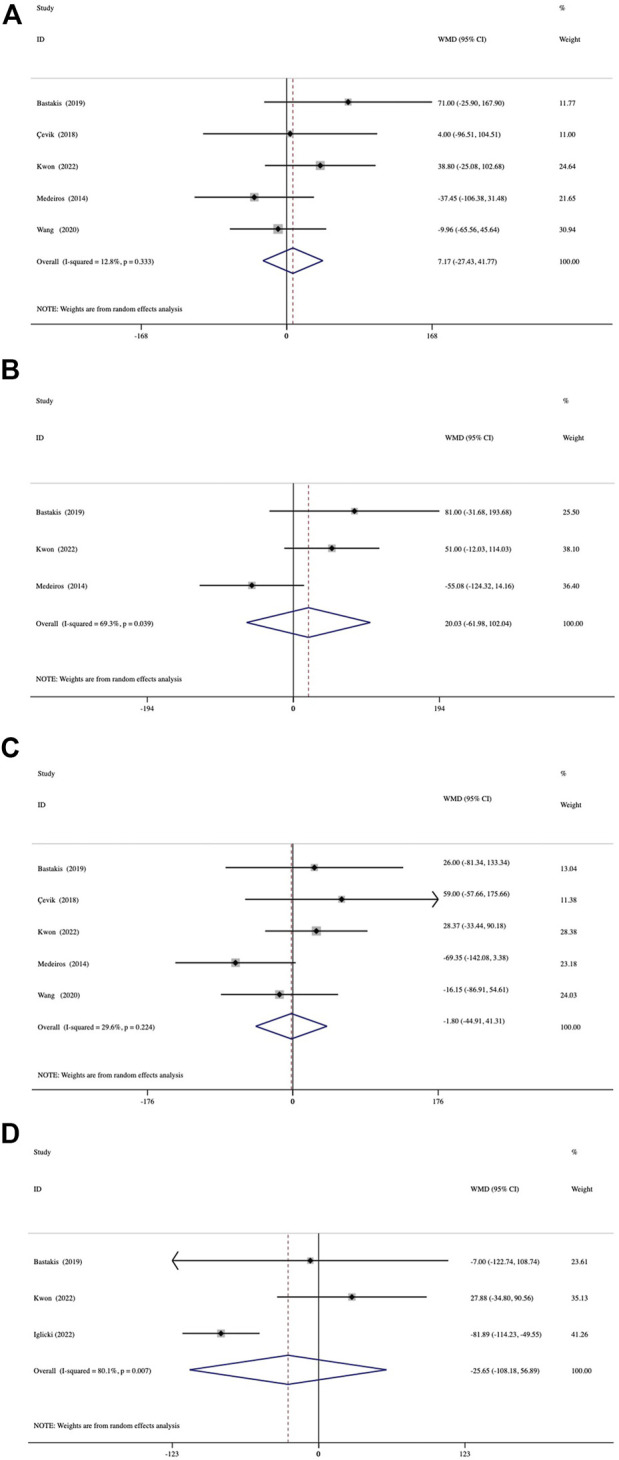
Forest plots of the comparison of mean CMT (central macular thickness) improvement between the vitrectomized and nonvitrectomized groups **(A)** 1 month, **(B)** 3 months, **(C)** 6 months, and **(D)** 12 months.

Both groups showed significant reductions in CMT at 1, 3, 6, and 12 months (all, *p* < 0.001) (Supplementary Table S2). A comparison of the mean reduction in CMT at different follow-up endpoints from baseline between the groups is shown in Figure 3. The assessment of 1-month reduction in CMT between the groups was based on data from 5 studies. The pooled results demonstrated no significant difference by 7.17 μm (95% CI, −27.43 to 41.77; *p* = 0.685) (Figure 3A). At 3 months, analysis of data from 3 studies showed a mean difference of 20.03 μm (95% CI, −61.98 to 102.04; *p* = 0.632) (Figure 3B). A comparison of 5 studies at 6 months was conducted, which presented a mean difference of −1.80−μm (95% CI, −44.91 to 41.31; *p* = 0.935) (Figure 3C). In 3 studies with a 12-month follow-up duration, the mean between-group differences in reduction in CMT was -25.65 μm (95% CI, −108.18 to 56.89; *p* = 0.542) (Figure 3D). No significant inter-study heterogeneity was found between the studies at 1 month (I^2^ = 12.8%, *p* = 0.333) or 6 months (I^2^ = 29.6%, *p* = 0.224).

### 3.5 Duration of dexamethasone action and the number of injections

**FIGURE 4:**
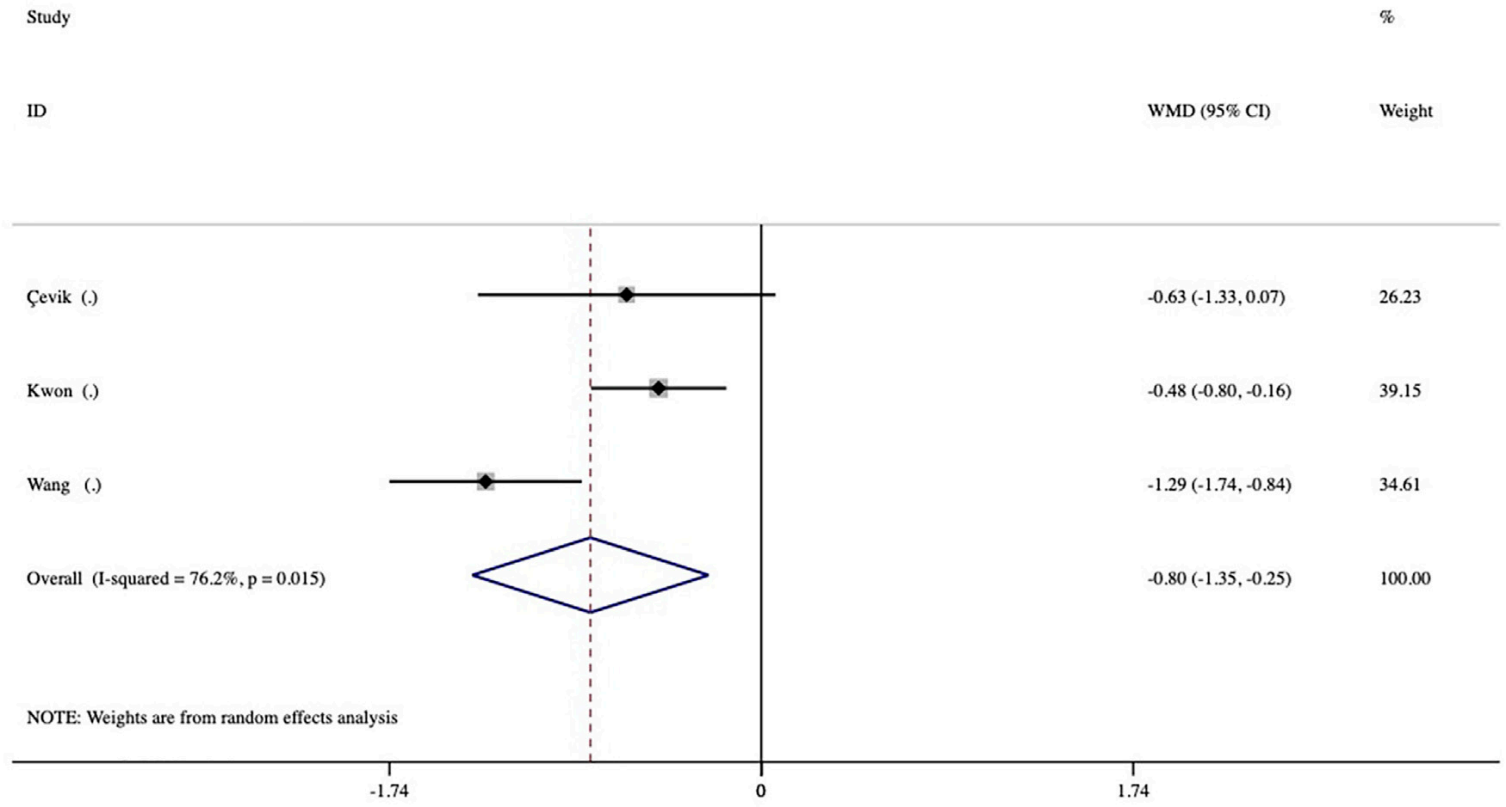
Forest plots of the comparison of the mean duration of action (intervals of macular edema recurrence) between the vitrectomized and nonvitrectomized groups.

**FIGURE 5:**
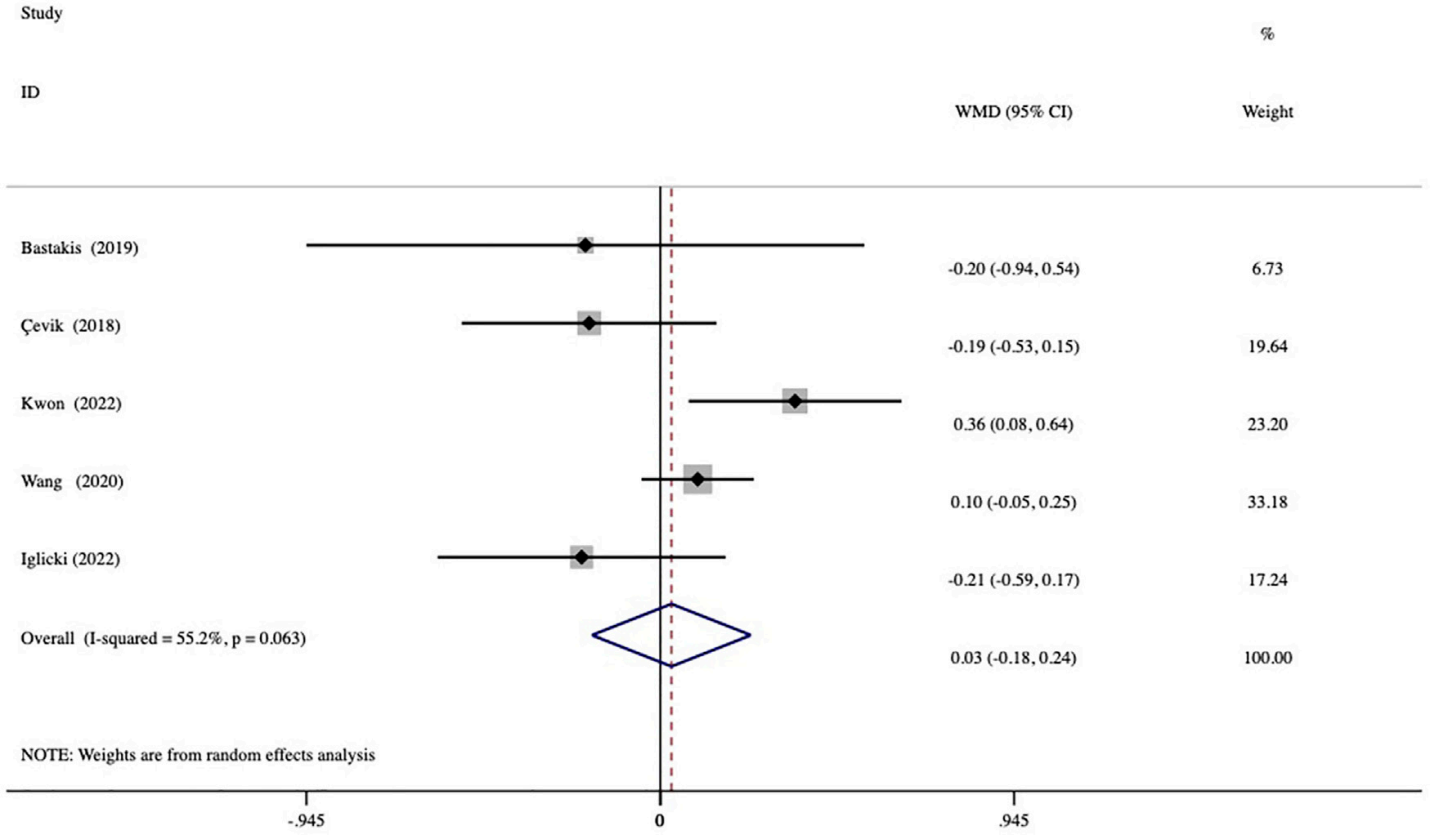
Forest plots of the comparison of the mean number of injections between the vitrectomized and nonvitrectomized groups during the follow-up period.

The duration of action during the first DEX implantation (interval of macular edema recurrence) was reported in 3 studies. The duration of action was significantly shorter in the vitrectomized group than in the nonvitrectomized group with an average difference of -0.80 months (95% CI, −1.35 to −0.25; *p* = 0.005) (Figure 4). Analysis of the mean number of DEX intravitreal injections over the follow-up period included 5 studies and no significant between-group differences in the number of injections was detected, with a mean of 0.03 times (*p* = 0.784) (Figure 5).

### 3.6 Quality assessment

All included studies were estimated as being of fair quality with a moderate risk of bias (score range, 15–18 points). In the assessment of BCVA, possible publication bias was detected by inspection of the funnel plot (Supplementary Figure S1A) and Egger test (*p* = 0.001). However, studies included in the CMT assessment presented no possible publication bias, as indicated by the funnel plot (Supplementary Figure S1B) and Egger test (*p* = 0.597). A considerable level of inter-study heterogeneity was detected in the comparison of BCVA gains at 12 months and reductions in CMT at 3 and 12 months. The sensitivity analysis indicated that the pooled results were stable and did not significantly change by eliminating any single study.

### 3.7 Safety

**FIGURE 6:**
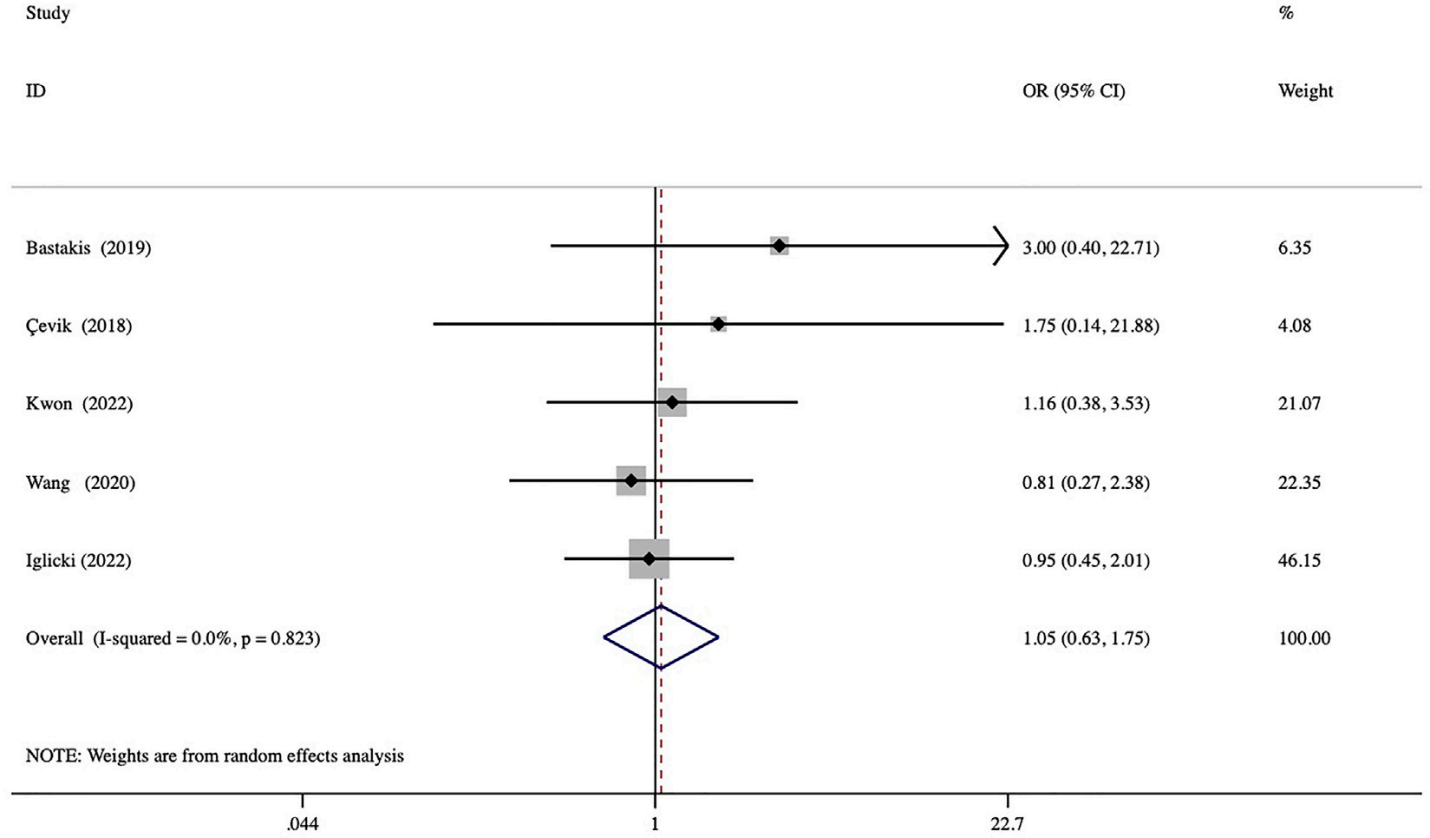
Forest plots of the comparison of the rate of elevated IOP (intraocular pressure) between the vitrectomized and nonvitrectomized groups.

No serious ocular and systematic AEs associated with DEX implantation were observed in any of the included studies. Among the several AEs reported, elevated IOP was the most frequent, and most patients were satisfactorily controlled with IOP-lowering drugs or observation. A comparison of the rates of elevated IOP showed no significant between-group differences (odd ratio = 1.05, *p* = 0.844) (Figure 6). Three studies reported cases of cataract formation or progression (Çevik et al., 2018; Iglicki et al., 2022; Kwon and Park, 2022). In addition, other minor AEs associated with injections were conjunctival hemorrhage, mild ocular pain, local hyperemia, and foreign body sensation.

## 4 Discussion

This meta-analysis was the first to comprehensively compare the efficacy and safety of intravitreal DEX implantation between vitrectomized and nonvitrectomized eyes with DME. The pooled results demonstrated no significant difference in BCVA gains and reductions in CMT at 1, 3, 6, and 12 months, indicating similar efficacy between the groups. However, the duration of action of the intravitreal DEX implant in the vitrectomized eyes was significantly shorter than that in the nonvitrectomized eyes. In addition, we found no significant between-group differences in terms of the mean number of required injections during the same follow-up period. Regarding safety data, the rate of a high IOP also was not significantly different between the groups.

The similar efficacy of the DEX intravitreal implant in the 2 groups could mainly be attributed to its sustained-release property. This biodegradable device was developed to slowly release DEX within 6 months. Theoretically, its efficacy should not be significantly affected by the microenvironment in the vitreous cavity. A study in rabbits showed a similar pharmacokinetic of the DEX intravitreal implant in nonvitrectomized and vitrectomized rabbit eyes, with DEX remaining for at least 31 days in both groups (Chang-Lin et al., 2011). The effectiveness and safety of the DEX implant in vitrectomized eyes with DME and ME associated with other retinal diseases, such as retinal vein occlusion and uveitis have been well demonstrated (Adán et al., 2013; Novais et al., 2016; Rezkallah et al., 2018).

Moreover, vitrectomy may alter the levels of some cytokines. Several studies have reported that angiogenesis-related factors, such as VEGF, hepatocyte growth factor, angiopoietin-2, and erythropoietin, were reduced *via* vitrectomy in eyes with proliferative diabetic retinopathy (Yoshida et al., 2010; Yoshida et al., 2012). However, some proinflammatory cytokines, such as monocyte chemoattractant protein-1 and interleukin-6, increase after vitrectomy in patients with proliferative diabetic retinopathy (Yoshida et al., 2015). Monocyte chemoattractant protein-1 has been reported to be a contributing factor to postoperative DME in vitrectomized eyes. Therefore, the anti-inflammatory effect of DEX implants may offset some of the effects of the increased rate of drug clearance and may be a beneficial therapy for vitrectomized eyes with DME.

This meta-analysis showed that the vitrectomized group presented a significantly shorter duration of action than the nonvitrectomized group. An *in vitro* experiment showed that DEX diffused 4 times faster through a saline solution than through a vitreous solution (Gisladottir et al., 2009). A retrospective, multicenter study also demonstrated significant shorter mean rejection intervals in baseline vitrectomized groups than in nonvitrectomized groups (5.2 months *versus* 6.9 months) (Rezkallah et al., 2018). However, the authors considered that a nearly 1-month difference between groups may not be clinically relevant. Given the small average duration difference of 0.8 months (<1 month) and nearly equal number of required injections during the same follow-up period in this meta-analysis, we still believe that DEX implantation was similarly effective in nonvitrectomized and vitrectomized eyes with DME. Nevertheless, well-designed prospective trials, especially randomized controlled trials, are required to confirm these findings.

No severe AEs were observed in any of the included studies. Increased IOP and cataract development were the most frequently reported AEs among the eligible trials. Most IOP increases could be controlled with topical IOP-lowering medications. We compared the rates of increased IOP between the vitrectomized and nonvitrectomized eyes. The pooled results suggested that the risk of increased IOP did not increase after vitrectomy, which is consistent with most findings of previous studies. Additionally, Kwon and Park reported that the maximal average IOP presented 1 month earlier in the vitrectomized eyes than in the non-vitrectomized eyes, although they detected no significant differences between the groups in the prevalence of increased IOP (Kwon and Park, 2022). DEX implants should be used cautiously in eyes with clear lenses and elevated IOP.

This meta-analysis has several limitations. First, our final analysis included a limited number of studies that were all retrospective in design. Second, all included trials were estimated as being of fair quality for having moderate risk of bias. Lastly, possible publication bias was detected in the BCVA data analysis.

## 5 Conclusion

In conclusion, our analysis showed no significant differences in anatomical and functional improvement between vitrectomized and nonvitrectomized eyes with DME treated with a DEX implant. The safety profile of the DEX intravitreal implant was well-balanced in both groups. Thus, the intravitreal DEX implant could be considered an effective and safe alternative in vitrectomized eyes for treatment of patients with DME.

## Data Availability

The original contributions presented in the study are included in the article/Supplementary Material, further inquiries can be directed to the corresponding author.

## Abbreviations

AEs, adverse events; CMT, central macular thickness; DEX, dexamethasone; DME, diabetic macular edema; DR, diabetic retinopathy; ME, macular edema.
