# Supplementary material for: Efficacy and safety of the dexamethasone implant in vitrectomized and nonvitrectomized eyes with diabetic macular edema: A systematic review and meta-analysis
Source: Front Pharmacol. 2022 Dec 1;13:1029584. doi: 10.3389/fphar.2022.1029584 (PMC9751612; doi:10.3389/fphar.2022.1029584)
Supplement: Supplementary file 1 [file DataSheet2.PDF]

Supplementary material

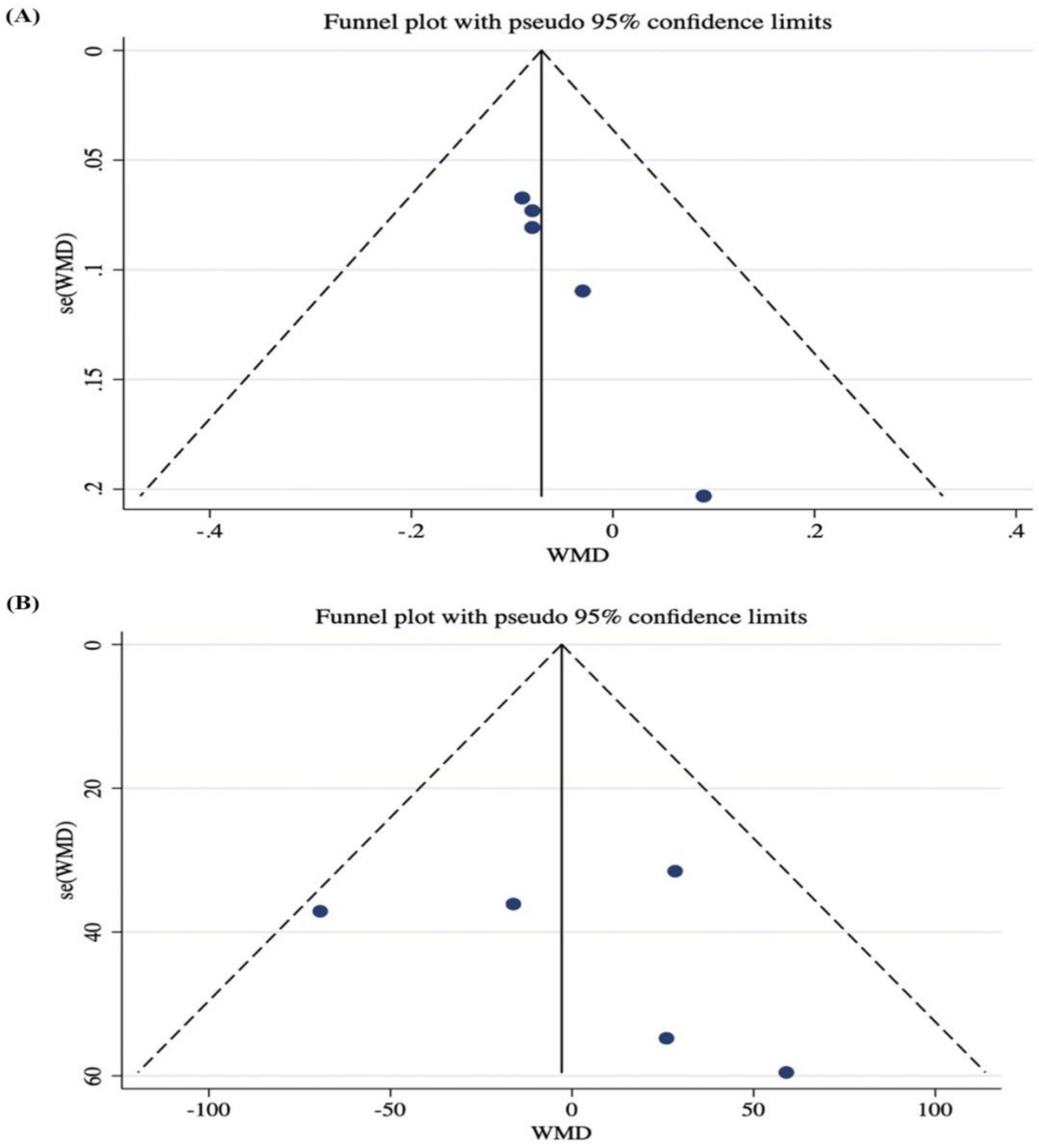

Figure S1. Funnel plots of the comparison of improvement in **(A)** BCVA (best-corrected visual acuity), and **(B)** CMT (central macular thickness) at 6 months between vitrectomized and nonvitrectomized groups.

Table S1. Study characteristics of the seven trials in the meta-analysis.

Authors	Year	Location	Study design	Sample eyes	Sex (M/F)	Mean age (y)	Mean number of DEX implants	Follow-up [months]	Cases of elevated IOP	Duration of action	Downs & Black Score
Bastakis et al	2019	Greece	retrospective	V: 10<br>NV: 8	V: 5/5<br>NV:3/4	V: 64<br>NV: 76	V: 2.5±0.85<br>NV: 2.7±0.76	18	V: 5<br>NV: 2	N/A	17
Çevik et al	2018	Turkey	retrospective	V: 9<br>NV: 31	N/A	V: 60.4 ± 9.2<br>NV: 63.1 ± 8.1	V: 1.2±0.4<br>NV: 1.39±0.62	V: 15.8±9.3 (6–36)<br>NV: 15.8±9.3 (6–36)	V: 1<br>NV: 2	V: 4.08±1.03<br>NV: 4.71±0.51	16
Kwon et al	2022	Korea	retrospective	V: 22<br>NV: 99	V: 10/12<br>NV: 52/47	V: 57.82±9.37<br>NV: 59.3±8.68	V: 3.41±0.67<br>NV: 3.06±0.68	12	V: 5<br>NV: 20	V: 3.59±0.67<br>NV: 4.07±0.75	18
Medeiros et al	2014	Spain	retrospective	V: 24<br>NV: 34	V: 17/7<br>NV: 22/12	V: 62.42±7.16<br>NV: 64.41±13.60	1	6	N/A	N/A	15
Wang et al	2020	Taiwan	retrospective	V: 27<br>NV: 43	V: 20/7<br>NV: 28/13	V: 59.13 ± 9.81<br>NV: 61.14 ± 10.85	V: 1.59±0.33<br>NV: 1.49±0.28	6	V: 7<br>NV: 13	V: 3.52±0.78<br>NV: 4.81±1.14	17
Bonnin et al	2015	France	retrospective	V: 10<br>NV: 29	22/12	63 ± 12	1	4	8	N/A	17
Iglicki et al	2022	Multicenter	retrospective	V: 106<br>NV: 130	V: 57/49<br>NV: 64/56	V: 70.43±13.91<br>NV: 68.82±13.63	V: 3.33±1.82<br>NV: 3.54±0.97	V: 25.57±11.80<br>NV: 24±0.0	V: 14<br>NV: 18	N/A	16

M, male; F, female; DEX, dexamethasone; IOP, intraocular pressure; V, vitrectomized; NV, nonvitrectomized N/A, not available.

Table S2. BCVA (logMAR) and CMT (μm) outcomes in vitrectomized eyes and nonvitrectomized eyes.

	Vitrectomized eyes		Nonvitrectomized eyes		Intergroup comparison of improvement
	Mean (95% CI)	p Value	Mean (95% CI)	p Value	
BCVA gains					
1 month	-0.22(-0.30 to -0.13)	p<0.001	-0.16(-0.23 to -0.09)	p<0.001	p=0.088
3 months	-0.21(-0.32 to -0.10)	p<0.001	-0.18(-0.30 to -0.06)	p<0.001	p=0.472
6 months	-0.22(-0.32 to -0.12)	p<0.001	-0.16(-0.25 to -0.06)	p=0.001	p=0.066
12 months	-0.17(-0.23 to -0.10)	p<0.001	-0.12(-0.24 to 0.00)	p=0.059	p=0.486
CMT reductions					
1 month	-197.79(-236.56 to -159.03)	p<0.001	-202.18(-250.62 to -153.75)	p<0.001	p=0.685
3 months	-201.58(-252.24 to -150.91)	p<0.001	-211.12(-254.04 to -171.20)	p<0.001	p=0.632
6 months	-169.90(-208.92 to -130.88)	p<0.001	-177.43(-236.96 to -117.91)	p<0.001	p=0.935
12 months	-247.52(-384.32 to -110.72)	p<0.001	-240.26(-298.55 to -181.97)	p<0.001	p=0.542

BCVA, best-corrected visual acuity; CMT, central macular thickness.
